# Prognostic Value of Peripheral Blood Lymphocyte Telomere Length in Gynecologic Malignant Tumors

**DOI:** 10.3390/cancers12061469

**Published:** 2020-06-04

**Authors:** Kamrunnahar Shanta, Kentaro Nakayama, Masako Ishikawa, Tomoka Ishibashi, Hitomi Yamashita, Seiya Sato, Hiroki Sasamori, Kiyoka Sawada, Sonomi Kurose, Hossain Mohammad Mahmud, Sultana Razia, Kouji Iida, Noriyoshi Ishikawa, Satoru Kyo

**Affiliations:** 1 Department of Obstetrics and Gynecology, Faculty of Medicine, Shimane University, Izumo 693-8501, Japan; kamrunnahar.vet@gmail.com (K.S.); m-ishi@med.shimane-u.ac.jp (M.I.); tomoka@med.shimane-u.ac.jp (T.I.); meme1103@med.shimane-u.ac.jp (H.Y.); sato_seiya9534@yahoo.co.jp (S.S.); sasamori@med.shimane-u.ac.jp (H.S.); kiyoka-s@med.shimane-u.ac.jp (K.S.); kurose.s@med.shimane-u.ac.jp (S.K.); likhon.vet@gmail.com (H.M.M.); raeedahmed@yahoo.com (S.R.); iida@med.shimane-u.ac.jp (K.I.); satoruky@med.shimane-u.ac.jp (S.K.); 2 Department of Organ Pathology, Shimane University Faculty of Medicine, Izumo 693-8501, Japan; kanatomo@med.shimane-u.ac.jp

**Keywords:** relative lymphocyte telomere length, ovarian cancer, cervical cancer, endometrial cancer, overall survival, progression-free survival

## Abstract

*Background:* Lymphocyte telomere length is strongly correlated with patient prognosis in several malignant tumor types and is thought to be related to tumor immunity. However, this correlation has not been studied in gynecological cancers. We determined the prognostic significance of peripheral blood lymphocyte telomere length in gynecologic cancers. *Methods:* Telomere length of lymphocytes from patients with gynecological malignant tumors (ovarian cancer (OC), *N* = 72; cervical cancer (CC), *N* = 63; endometrial cancer (EC), *N* = 87) was examined by quantitative reverse-transcription PCR of isolated mononuclear cells. Kaplan–Meier and Cox proportional hazard analyses were used to determine the association between lymphocyte telomere length and clinicopathological factors. *Results:* The overall survival (OS) and progression-free survival (PFS) of patients were based on the dichotomized lymphocyte telomere length using the median as a threshold (OC: 0.75, CC: 1.94, and EC: 1.09). A short telomere length was significantly correlated with residual tumors (≥1 cm) in OC and with advanced stage (III and IV) of CC. In OC and CC, patients with shorter relative lymphocyte telomere length (RLT) had significantly poorer OS and PFS than patients with longer RLT (*p* = 0.002, *p* = 0.003, and *p* = 0.001, *p* = 0.001, respectively). However, in EC, RLT was not significantly associated with OS or PFS (*p* = 0.567 and *p* = 0.304, log-rank test). Multivariate analysis showed that shorter RLT was a significant independent prognostic factor of PFS and OS for OC (*p* = 0.03 and *p* = 0.04, respectively) and CC (*p* = 0.02 and *p* = 0.03, respectively). *Conclusions:* Patients with OC and CC with shorter lymphocyte telomeres have significantly reduced survival; therefore, the peripheral blood lymphocyte telomere length is a prognostic biomarker in OC and CC.

## 1. Introduction

Telomeres, the terminal structures of chromosomes, are composed of long, repetitive 6-base pair nucleoproteins. Their vital role is to maintain chromosomal integrity and genomic stability by blocking the end-to-end fusion of chromosomes, nuclease degradation, and incomplete replication during cell division [1,2]. In human somatic cells, the average length of telomeres is 10–15 kb, and telomeric DNA is shortened during each cell division by approximately 50–200 base pairs [3,4]. When the telomere length reaches a critical point because of this progressive shortening, replicative senescence is triggered, resulting in cell-cycle arrest or apoptosis [5,6]. Abnormal shortening of telomeres causes genomic instability in the form of chromosomal rearrangement or abnormal chromosome numbers, which can trigger carcinogenesis [7,8]. Short telomeres are more prevalent in tumor tissues than in adjacent non-malignant tissue [9]. Short telomeres in peripheral blood cell DNA are associated with an increased risk of various human malignant tumors [10,11] including ovarian cancer (OC) [12], bladder cancer [13], gastric adenocarcinoma [14], colorectal cancer [15], and pancreatic cancer [16]. However, increased peripheral blood cell telomere length can be a predisposing factor for developing other cancers [17]. Decreased telomere length in leukocytes is significantly associated with poor prognosis in gastric cancer [18], glioma [19], colorectal cancer [20], and oral squamous cell carcinoma [21], whereas increased lymphocyte telomere length is associated with a risk of developing breast cancer [22], lymphocyte leukemia [23], and renal cell carcinoma [24]. No studies have appraised the prognostic value of lymphocyte telomere length in gynecological malignant tumors. Among the gynecological cancers, OC is the seventh most common malignant tumor in women worldwide [25]. In 2019, there were nearly 22,530 newly diagnosed cases of OC and 13,980 associated deaths in the United States [26]. Previous studies on OC have shown that telomeres are shorter with increased telomerase activity in malignant epithelial ovarian tumor and that the shorter telomeres of peripheral blood leucocyte were associated with increased risk of ovarian adenocarcinoma [27]. However, a recent study showed that shorter telomere was associated with high risk of both familial and sporadic OC [12]. Despite the availability of therapeutic regimens, the prognosis of patients with OC remains poor, with a five-year survival rate below 50% in nearly all countries. Cervical cancer (CC) is another major cause of mortality, ranking globally as the fifth most common malignant neoplasm in women [28]. The reason for the increased mortality associated with CC is unclear, but common genetic mutations appear to be a significant motivating force in tumor growth [29]. Finally, the incidence of endometrial cancer (EC) has been increasing, and EC now ranks as the sixth most common gynecologic malignant tumor [30]. Typically, patient outcomes are evaluated by considering several factors including tumor grade, extent of tumor resection, tumor metastasis, cancer stage, and patient age; however, significant discrepancies exist between characteristically equivalent patients. This makes it difficult to identify effective molecular biomarkers for prognostic prediction of gynecological malignant tumors.

A few scattered studies have evaluated blood-based intermediate biomarkers such as DNA repair capacity and telomere length for predicting the risk of developing various cancers [17], but no studies have evaluated the prognostic significance of lymphocyte telomere length in gynecological cancer patients. In the present study, we conducted reproducible quantitative PCR to measure the lymphocyte telomere length and evaluate its prognostic significance in patients with gynecological cancer prior to undergoing surgical resection.

## 2. Results

### 2.1. Relationship between Telomere Length and Clinicopathological Factors

The demographic and clinical characteristics of the included patients with gynecological cancer are summarized in Table 1. Peripheral blood lymphocyte telomere length was obtained using quantitative reverse-transcription PCR (qRT-PCR), and the optimal cutoff for the relative lymphocyte telomere lengths (RLTs) to dichotomize patients into the long or short groups was determined (OC: 0.75, CC: 1.94, and EC: 1.09). Spearman’s correlation analysis showed that RLTs in all three cancer categories were negatively correlated with age (Figure S1). We also found that the correlation between FIGO (International Freedom of Gynecology and Obstetrics) stages (I, II, III, and IV) and lymphocyte telomere length was significantly higher in patients with advanced stages III and IV than early stages I and II (60.71% vs. 34.09% in OC, *p* = 0.027; 77.77% vs. 33.33% in CC, *p* = 0.001; 75% vs. 38.80% in EC, *p* = 0.001). No significant correlation was found between tumor grade and telomere length in OC (*p =* 0.69) or EC (*p =* 0.17), and analysis of the CC tumor grade was not performed. The correlation of myometrial invasion and menopause with telomere length was evaluated in patients with EC, and no significant correlation was found; however, lymph vascular space and lymph node metastasis were both significantly correlated with RLT in patients with EC (*p* = 0.015; *p* = 0.008). Residual tumor status (≥1 cm) in patients with OC was significantly correlated (*p* = 0.028) with a short RLT. There was no significant correlation between the RLT and histological subtype (*p =* 0.493).

### 2.2. Effects of Lymphocyte Telomere Length on Progression-Free Survival (PFS)

**Figure 1:**
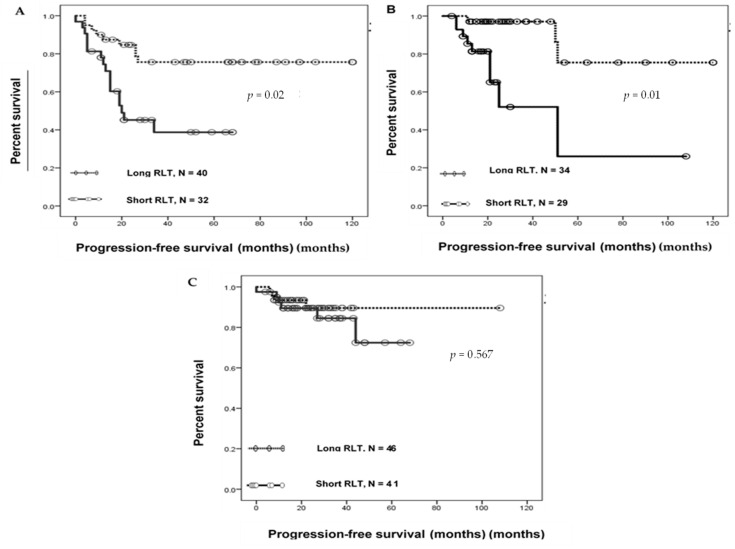
Relative lymphocyte telomere length (RLT) and progression-free survival in gynecological cancers. Kaplan–Meier progression-free survival analysis in (**A**) patients with ovarian cancer with short RLT (solid line, *N* = 32) and long RLT (dotted line, *N* = 40); (**B**) patients with cervical cancer with short RLT (solid line, *N* = 29) and long RLT (dotted line, *N* = 36); and (**C**) based on the relative lymphocyte telomere length in patients with endometrial cancer.

We examined whether the RLT affected PFS (Figure 1A–C). To predict PFS, the absolute value of RLT (Table S1) was dichotomized by the median value, which subdivided the patients into those with short and long RLT [14]. Kaplan–Meier curve analysis indicated that in both OC and CC, patients with short RLTs had significantly lower PFS than those with long RLTs (log-rank *p =* 0.002 for OC and *p =* 0.001 for CC). However, PFS in EC showed no significant correlation with RLT (log-rank *p =* 0.567) (Figure 1C). The median PFS of patients with short and long RLTs was 15 and 47.5 months for OC, 17 and 25.5 months for CC, and 12 and 22 months for EC, respectively. Univariate Cox proportional hazard regression analysis indicated that for OC, FIGO stages III and IV (log-rank test, *p* < 0.001), tumor grades G2 and G3 (log-rank test, *p =* 0.04), residual tumor ≥1 cm (log-rank test, *p* < 0.001), and short RLT (log-rank test, *p =* 0.004) were correlated with a shorter PFS (Table 2); patient age and histology had no effect on PFS (*p =* 0.96 and *p =* 0.069, respectively). Multivariate analysis showed that the FIGO stage and RLT were independent variables for the PFS of patients with OC. This analysis confirmed that the effects of FIGO stage and RLT on PFS were significant in OC (log-rank test, *p =* 0.001 and *p =* 0.03, respectively). For CC (Table 3), univariate analysis revealed that FIGO stages IIB, III, and IV (log-rank test, *p =* 0.023) and short RLT (log-rank test, *p =* 0.005) were correlated with a shorter PFS; patient age and histology had no effect on PFS (*p =* 0.56 and *p =* 0.87, respectively). Multivariate analysis also showed that RLT was an independent variable for PFS and confirmed the influence of RLT on PFS in CC (log-rank test, *p =* 0.02).

### 2.3. Effects of Lymphocyte Telomere Length on Overall Survival (OS)

**Figure 2:**
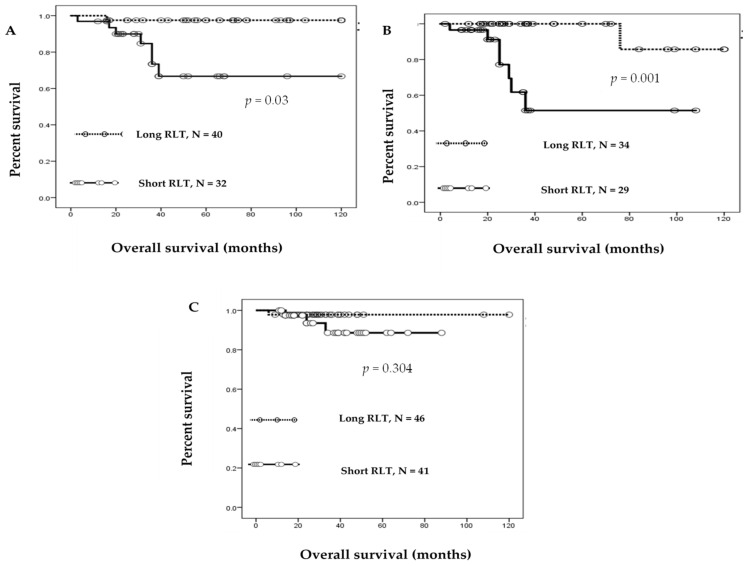
Relative lymphocyte telomere length (RLT) and overall survival of patients with gynecological cancer. Kaplan–Meier overall survival analysis in (**A**) patients with ovarian cancer with short RLT (solid line, *N* = 32) and long RLT (dotted line, *N* = 40); (**B**) patients with cervical cancer with short RLT (solid line, *N* = 29) and long RLT (dotted line, *N* = 36); and (**C**) based on the relative lymphocyte telomere length in patients with endometrial cancer.

The prognostic significance of RLT was determined by the Kaplan–Meier estimation of OS (Figure 2). Among the 72 patients with OC diagnosed at stages I–IV, 32 with a short RLT had worse OS than those with a long RLT (log-rank test, *p =* 0.003); univariate analysis demonstrated that FIGO stages III and IV (log-rank test, *p =* 0.029), residual tumors ≥1 cm (log-rank test, *p =* 0.001), and short RLT (log-rank test, *p* = 0.01) were correlated with OS (Table 4); patient age and histology were not significantly associated with OS (log rank test, *p* = 0.82, *p =* 0.36, respectively). Multivariate analysis showed that residual tumor and RLT were independent variables for OS and confirmed their significant association with worse OS in OC (*p* = 0.003, *p* = 0.04, respectively). For the 63 patients with CC diagnosed at stages I–IV, 29 with a short RLT had worse prognosis than those with a long RLT (log-rank test, *p* = 0.001) (Figure 2B); short RLT was found to be significantly associated with poor prognosis in both univariate and multivariate analyses (*p* = 0.01, *p* = 0.03, respectively), which showed RLT as an independent variable for OS. However, the FIGO stage was found to be significantly correlated with OS by univariate analysis (*p* = 0.03), but not by multivariate analysis (*p* = 0.51) (Table 5). No meaningful correlation between RLT and OS was found by Kaplan–Meier analysis of patients with EC (log-rank test, *p* = 0.304).

### 2.4. Comparison of Lymphocyte Relative Telomere Length before and after Treatment

**Figure 3:**
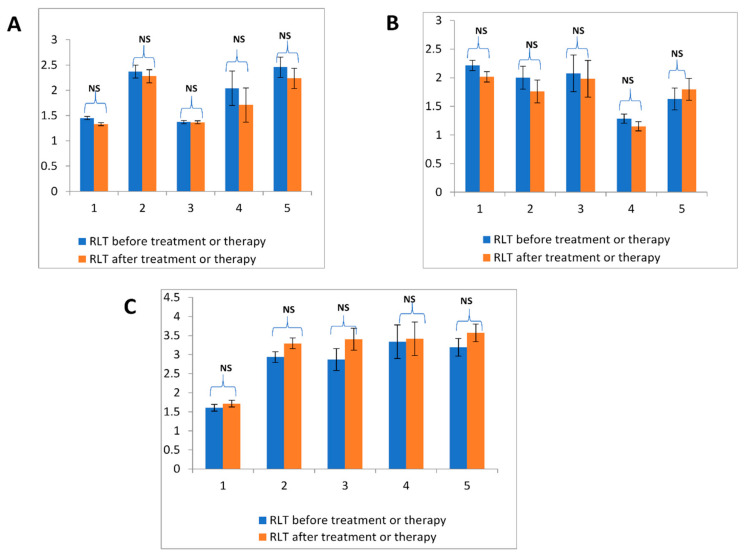
Comparison of relative blood lymphocyte telomere length in patients before and after treatment or therapy. Relative lymphocyte telomere length (RLT) before (blue) and after (red) surgery in (**A**) ovarian cancer, (**B**) cervical cancer, and (**C**) endometrial cancer. Statistical significance was determined by the Student’s *t* test, *p* < 0.05 was considered significant. NS; not significant.

In this study, a few blood samples (10.36%) were collected from patients after surgery and/or chemo- or radiotherapy. To determine whether the time of sample collection affected the peripheral RLT, we analyzed blood samples from 5% (*n* = 15) of patients both before and after surgery. In all cancer-types, peripheral RLT did not change significantly following treatment (Figure 3).

## 3. Discussion

Peripheral lymphocyte telomere length has been established as a prognostic biomarker in different malignant tumors [31]. In this study, we determined the significant prognostic value of lymphocyte telomere length in patients with gynecological cancer. Patients with OC and CC with short RLT had worse PFS and OS. Short leukocyte telomeres are associated with poor OS and PFS in glioma [19], colorectal cancer, and gastric cancer [18,20]. In addition, several epidemiological studies have established that blood leukocyte telomere is closely associated with the risk of development of gynecological cancer [32].Two studies have shown increased association between short leukocyte telomere length and risk of OC [12,27], while one study using blood samples indicated that short telomere length is associated with malignancies of OC [12]. These findings as well as those from the present study, support that cancer patients with short lymphocyte telomeres have poorer outcomes because of telomere attrition [33]. Here, we demonstrated that telomere length is associated with the clinical stage in gynecological cancers, consistent with the results of a previous study [34] and strengthening the evidence that short telomeres are positively associated with advanced carcinogenic stages. Notably, as described above, in renal cell carcinoma, breast cancer, and prostate cancer, long rather than short telomeres are associated with poor prognosis [35]. This discrepancy suggests that the association between telomere length and cancer prognosis is organ-specific and depends on the molecular mechanisms of tumorigenesis. Although the mechanisms underlying the present findings require further investigation, it is clear that blood telomere dysfunction plays an important role in carcinogenesis.

According to the above information, it was expected that RLT would be correlated with survival in EC. However, no significant association was found between RLT, as measured in peripheral blood lymphocytes, and the OS or PFS of 87 patients with EC. The endometrium is the primary target organ of ovarian steroid hormone, and the cell cycle is strictly regulated by these hormones. The results of this study are similar to those of a previous study investigating the relationship between telomere length in the peripheral blood and breast cancer survival outcomes, which revealed no significant correlation between telomere length and OS [36]. Breast, prostate, and endometrial cancers are generally related to hormones, whereas malignant neoplasms associated with short telomere length such as gastric and colorectal cancers may not depend on hormone regulation. These studies suggest that hormone-dependent cancer might be linked to telomere length regulation by sex hormones.

Cancer immunity involves a combination of tumor-infiltrating lymphocytes and circulating peripheral blood lymphocytes [37]. Particularly, elevated levels of circulating cytotoxic CD8 + T cells in the tumor microenvironment are associated with prolonged PFS and OS [38], which is strongly correlated with long telomere length. In contrast, increased numbers of CD4 + T cells in peripheral blood mononuclear cells were significantly related to poor prognosis in gastric cancer with short telomere length [18]. In this study, we also found that short blood lymphocyte telomere length was associated with poor prognosis of OC, and CC with long RLT may be strongly correlated with increased CD8 + T cells and an effective antitumor response (Figure S2). Moreover, expression of the cytokines IL-2, IL-15, and IL-21 promotes the activation of CD8 + T cells and is associated with a better prognosis than that with IL-10 promotion of regulatory CD4 + T cells [18,39,40]. The massive expansion of CD8 + T cell actively depends on interferon alpha (IFN*α*), which improves prognosis by causing cancer cell cycle arrest, whereas CD4 +, which is related to IFN-*γ*, is associated with poor prognosis in patients with cancer (Figure S2) [41]. Furthermore, cancer cells utilize immune escape mechanisms such as the programmed cell death-1(PD-1)/PD-1 ligand (PD-L1) pathway [42,43]. Various forms of cancer immune therapy have been established to improve CD8 + T cell-induced anti-tumor immunity such as immune checkpoint blockade. Immune checkpoint blockade stimulates the immune system by PD-1- or PD-L1-blocking antibodies. Infiltration of CD8 + T cells in renal carcinoma was associated with the response to upregulation of PD-1 antibodies with insertion or deletion-derived neoantigens [44]. This suggests that the increased number of CD8+ T cells in tumor cells activate immune checkpoint inhibitors [45,46]. Based on these results and those of previous studies, we hypothesized that long telomeres with increased CD8 + T cells may be effective as immune checkpoint inhibitors for treating patients with cancer (Figure S2). Further studies with immunostaining are needed to confirm these molecular expression predictions.

There were some limitations to this study. First, the hypothesis that lymphocyte telomere length is associated with host immunity is based on the findings of a previous study [33]. To confirm this hypothesis, further clinical investigations are required. Second, the small sample size used to analyze OS lacks high-quality confirmation, which is inherent to the observational study design. However, this study used a long follow-up period, triplicate measurements of PCR samples, and adjustment for confounders.

## 4. Materials and Methods

### 4.1. Patient Data Collection

Clinical patient data and follow-up data were collected through a retrospective review of medical archives and electronic medical records at Shimane University Hospital. Age, tumor stage, grade, residual tumor status, lymph node metastasis, histological categories, time of surgery, and time of recurrence or death were recorded. Tumor type and stage were assessed according to the World Health Organization and the International Federation of Gynecology and Obstetrics (FIGO), respectively [47]. The latest follow-up date was December 2018, and the follow-up duration was 120 months. OS was defined as the length of time from the date of diagnosis to death or last follow-up. PFS was defined as the length of time from surgery to the date of first clinical deterioration [48]. All patients included in this study provided written informed consent for the use of their clinical and pathological data. There were no age or cancer stage restrictions at recruitment.

### 4.2. Blood Samples

A total of 222 peripheral blood samples were collected from patients with gynecological cancer from the Department of Obstetrics and Gynecology at Shimane University Hospital. Among them, 199 patients (89.63%) had blood collected prior to surgical resection, whereas 23 samples (10.36%) were collected after surgery, chemotherapy, or radiotherapy. Among all patients, 72 had OC (range, 25–85 years), 63 had CC (range 29–79 years), and 87 had EC (range, 24–72 years). All patients were administered appropriate therapy at Shimane University Hospital between 2008 and 2018. Patients with OC were primarily treated with cytoreductive surgery and adjuvant platinum and taxane chemotherapy (carboplatin, paclitaxel; 175 mg/m^2^ or docetaxel; 70 mg/m^2^); all patients were administered 6–12 courses of this combination regimen. Patients with CC in stages I and II were treated with class II or class III radical hysterectomies with pelvic lymph node dissection. Stage I patients were treated with positive lymph node vascular space invasion, and all stage II patients underwent concurrent chemotherapy or radiotherapy. Patients with stages III and IV CC were treated with concurrent chemotherapy or radiotherapy alone. Patients who showed incomplete response to radiotherapy and recurrent tumors were treated with a variety of salvage chemotherapy agents (cisplatin, carboplatin, and paclitaxel). All patients with EC underwent surgery (total abdominal hysterectomy, modified radical hysterectomy, and bilateral salpingo-oophorectomy); those at a stage higher than IA and grade 1 underwent pelvic lymph node dissection and adjuvant platinum and taxane chemotherapy. Pelvic lymph node dissection and adjuvant chemotherapy were not performed in patients with stage 1A, EC without myometrial invasion, and grade 1/2 disease. Confirmation of patient eligibility was based on conventional morphological examination and availability of clinical follow-up data. Acquisition of blood samples and clinical information were approved by the institutional review board of Shimane University Hospital (approval no. 2004-0381).

### 4.3. DNA Extraction for Quantitative PCR

For DNA extraction, venous blood samples (5 mL) were drawn from each patient into a vacutainer tube containing anticoagulant (EDTA, heparin, ACD). Lymphocytes were collected using Lymphoprep^TM^ (AXIS-SHIELD PoC AS, Oslo, Norway), according to the manufacturer’s instructions. Next, DNA was extracted from the isolated lymphocytes with a QIAGEN DNase blood and tissue kit (Hilden, Germany) following the manufacturer’s instructions and stored at −80 °C until quantitative PCR analysis. DNA was quantified using a spectrophotometer (NanoDrop 2000; Thermo Fisher Scientific, Waltham, MA, USA) to ensure accuracy and concentration uniformity.

### 4.4. Measurement of Telomere Length by QRT-PCR

Relative lymphocyte telomere length was measured using qRT-PCR as previously described with some modification [49]. Briefly, in qRT-PCR, the ratio of the telomere copy number (T) to the single gene copy number (S) was determined for each sample using standard curves. The derived ratio (T/S) is proportional to the telomere length. Henceforth, the ratio of each sample was normalized to a calibrator DNA sample to standardize between each successive run. RLT value was then used during experimentation from this calibrated *T*/*S* (Formula: *T*/*S* = 2Δ*Ct*, where Δ*Ct* = *Ct* control − *Ct* telomere). PCR was performed in triplicate where the mean value was used. The reaction mixture of sample DNA was added to each 96-well PCR plate at a final volume of 20 μL, containing 20 ng of DNA, 2 × SYBR green master mix (Roche Diagnostics, Mannheim, Germany), and the appropriate primers. The primers (5′–3′) used for the telomere sequence were tel1 GGTTTTTGAGGGTGAGGGTGAGGGTGAGGGTGAGGGT and tel2 TCCCGACTATCCTATCCCATCCCTATCCCTATCCCTA, and those for the single-copy gene were 36b4u CAGCAAGTGGGAAGGTGTAATCC and 36b4d CCCATTCTATCATCAACGGGTACAA [50]. Gene-specific amplification was performed using the thermal cycler dice real-time system software TP800 (Takara, Shiga, Japan). The thermal cycling profile for both amplifications was as follows: one cycle at 95 °C for 10 min, followed by 40 cycles at 95 °C for 15 s, and then either 54 °C for 2 min (telomere) or 58 °C for 1 min (36B4). Real-time amplification of the telomere sequence and 36B4 were performed on separate 96-well plates. The reference DNA was applied to produce a standard curve for a range of 0.62–20 ng) to minor the PCR efficiency. Each plate also included a calibrator (blood from a healthy patient) in each triplicate run to assess the difference of the PCR plate. A negative control and standard curve were also used to confirm the specific amplification and assess the primer dimer artifacts. All investigators conducting this experiment were blinded to the end point of the experiment.

### 4.5. Statistical Analysis

Statistical analysis was performed using SPSS statistical software, version 21 (SPSS, Inc., Chicago, IL, USA). Spearman correlation analysis was used to assess the relationship between age and RLT. The Pearson correlation coefficient test was performed to examine the statistical difference between RLT and the clinical characteristics of the patient subgroups. Student’s *t*-tests (for comparison of two groups) or one-way analysis of variance was performed to compare RLTs of patients in different treatment conditions. The absolute value of RLT (Supplementary Table S1) was used as a category variable that was dichotomized as the median for OS and PFS [19]. Kaplan–Meier curves were strategized for survival analysis, and statistical significance was determined by the log-rank test. All data were censored when the patients died or for lack of follow-up. Multivariate prognostic analysis was performed using a Cox proportional hazard regression model to calculate the hazard ratio and 95% confidence interval for the association of clinicopathological variables and RLT on OS and PFS. *p <* 0.05 was considered statistically significant.

## 5. Conclusions

In summary, our findings indicate that a short telomere length of blood lymphocyte significantly affects the OS and PFS of patients with ovarian and cervical cancers, but is not associated with endometrial cancer that may involve immune dependent activities. Our results also suggest that lymphocyte telomere length is an important prognostic biomarker in cancer. Further studies are warranted to evaluate the molecular mechanism of immunosuppressive phenotype associated with short telomere length in a large sample for precise results.

## Figures and Tables

**Table 1:** Association of telomere length and clinicopathological factors in patients with ovarian, cervical, and endometrial cancer.

**Ovarian Cancer**				
**Factors**	**No. of cases (N)**	**Long RLT**	**Short RLT**	***p*** **-Value**
Age (years)		60.15(14.11)	69.53(10.71)	0.002 ^a^
Grade				
G1	15	9(60%)	6(40%)	0.69 ^b^
G2, G3	57	31(54.38%)	26(45.61%)	
FIGO stage				
I, II	44	29(65.90%)	15(34.09%)	0.027 ^b^
III, IV	28	11(39.28%)	17(60.71%)	
Histology				
Serous	35	17(48.57%)	18(51.42%)	0.49 ^b^
others	37	15(40.54%)	22(59.45%)	
Residual Tumor				
<1cm	54	34(62.96%)	20(37.03%)	0.028 ^b^
≥1cm	18	6(33.33%)	12(66.66%)	
**Cervical Cancer**				
**Factors**	**No. of cases**	**Long RLT**	**Short RLT**	***p*** **-Value**
Age (years)		48.73(13.93)	58.35(13.57)	0.033 ^a^
FIGO Stage				
I & IIa	45	30(66.66%)	15(33.33%)	0.001 ^b^
IIb, III, IV	18	4(22.22%)	14(77.77%)	
Histology				
SCC	33	16(48.48%)	17(51.51%)	0.36 ^b^
AC/ASC	30	12(40%)	18(60%)	
**Endometrial Cancer**				
**Factors**	**No. of cases**	**Long RLT**	**Short RLT**	***p*** **-Value**
Age (years)		61.19(13.08)	59.17(12.85)	0.021 ^a^
Grade				
GI	47	28(59.57%)	19(40.42%)	0.17^b^
G2, G3	40	18(45.00%)	22(55.00%)	
FIGO Stage				
I, II	67	41(61.19%)	26(38.80%)	0.004^b^
III, IV	20	5(25%)	15(75%)	
Histology				
Endometroid	72	34(47.22%)	38(52.77%)	0.96^b^
Others	15	7(46.66%)	8(66.66%)	
Lymph node metastasis				
Negative	78	45(57.69%)	33(42.30%)	0.008^b^
Positive	9	1(11.11%)	8(88.88%)	
Lymph vascular space				
Negative	48	31(64.58%)	17(35.41%)	0.015^b^
Positive	39	15(38.46%)	24(61.53%)	
Myometrial invasion				
a, b	65	30(46.15%)	35(53.84%)	0.75^b^
c	22	11(50%)	11(50%)	
Menopause				
Pre	30	16(53.33%)	14(46.66%)	0.40^b^
Post	57	32(56.14%)	25(43.85%)	

Notes: FIGO, International Freedom of Gynecology and Obstetrics; RLT, relative lymphocyte telomere length; SCC, squamous cell carcinoma; AC, adenocarcinoma; ASC, adenosquamous carcinoma. Data are the mean (SD) or *N* (%). *^a^*The *p*-values was calculated using a Spearman’s correlation analysis *^b^* The *p*-values was calculated using a Pearson chi-square test.

**Table 2:** Univariate and multivariate analyses of progression-free prognostic factors in patients with ovarian cancer.

Factors	Patients	Univariate Analysis			Multivariate Analysis		
		Hazard Ratio	95% CI	*p*-Value	Hazard Ratio	95% CI	*p*-Value
**Age**							
**<65**	33	0.98	0.45–2.14	0.96	NA	NA	NA
**≥65**	39						
**Grade**							
**G1**	15	4.39	1.04–18.67	0.04	1.24	0.22–6.86	0.8
**G2, G3**	57						
**FIGO stage**							
**I, II**	44	11.57	4.53–29.68	≤0.001	7.97	2.36–26.90	0.001
**III, IV**	28						
**Histology**							
**Serous**	35	0.47	0.21–1.06	0.06	NA	NA	NA
**Others**	37						
**Residual tumor**							
**<1 cm**	54	7.34	3.20–16.82	≤0.001	2.09	0.78–5.58	0.13
**≥1 cm**	18						
**RLT**							
**Long**	40	3.29	1.45–7.45	0.004	2.58	1.05–6.34	0.03
**Short**	32						

CI, confidence interval; FIGO, International Freedom of Gynecology and Obstetrics; RLT, relative lymphocyte telomere length.

**Table 3:** Univariate and multivariate analyses of progression-free prognostic factors in patients with cervical cancer.

Factors	Patients	Univariate Analysis			Multivariate Analysis		
		Hazard Ratio	95% CI	*p*-Value	Hazard Ratio	95% CI	*p*-Value
**Age**							
**<57**	34	1.4	0.44–4.68	0.56	NA	NA	NA
**≥57**	29						
**FIGO stage**							
**I, IIA**	15	3.77	1.19–11.92	0.023	1.95	0.56–6.78	0.28
**IIB, III, & V**	48						
**Histology**							
**SCC**	33	1.09	0.35–3.39	0.87	NA	NA	NA
**AC/ASC**	30						
**RLT**							
**long**	34	6.88	1.80–26.27	0.005	5.17	1.21–22.06	0.02
**Short**	29						

AC, adenocarcinoma; ASC, adenosquamous carcinoma; RLT, CI, confidence interval; FIGO, International Freedom of Gynecology and Obstetrics; RLT, relative lymphocyte telomere length; SCC, squamous cell carcinoma.

**Table 4:** Univariate and multivariate analyses of overall prognostic factors in patients with ovarian cancer.

Factors	Patients	Univariate Analysis			Multivariate Analysis		
		Hazard Ratio	95% CI	*p*-Value	Hazard Ratio	95% CI	*p*-Value
**Age**							
**<65**	35	1.17	0.28–4.92	0.82	NA	NA	NA
**≥65**	37						
**Grade**							
**G1**	15	9.33	1.145–76.17	0.33	NA	NA	NA
**G2, G3**	57						
**FIGO stage**							
**I, II**	44	5.96	1.19–29.77	0.02	0.12	0.07–2.04	0.14
**III, IV**	28						
**Histology**							
**Serous**	35	0.51	0.12–2.16	0.365	NA	NA	NA
**Others**	37						
**Residual tumor**							
**<1 cm**	54	33.72	4.07–279.15	0.001	124.53	5.19–298.53	0.003
**≥1 cm**	18						
**RLT**							
**Long**	40	12.44	1.15–102.23	0.01	16.29	1.02–259.67	0.04
**Short**	32						

CI, confidence interval; FIGO, International Freedom of Gynecology and Obstetrics; RLT, relative lymphocyte telomere length.

**Table 5:** Univariate and multivariate analyses of overall prognostic factors in patients with cervical cancer.

Factors	Patients	Univariate Analysis			Multivariate Analysis		
		Hazard Ratio	95% CI	*p*-Value	Hazard Ratio	95% CI	*p*-Value
**Age**							
**<57**	33	2.55	0.50–12.9	0.25	NA	NA	NA
**≥57**	39						
**FIGO stage**							
**I, IIA**	15	4.73	1.13–19.85	0.033	1.66	0.35–7.78	0.51
**IIB, III, & V**	48						
**Histology**							
**SCC**	33	1.5	0.26–4.21	0.94	NA	NA	NA
**AC/ASC**	30						
**RLT**							
**long**	34	14.57	1.77–119.84	0.01	11.26	1.17–107.86	0.03
**Short**	29						

AC, adenocarcinoma; ASC, adenosquamous carcinoma; RLT, relative lymphocyte telomere length; CI, confidence interval; FIGO, International Freedom of Gynecology and Obstetrics; SCC, squamous cell carcinoma.

## Supplementary Materials

The following are available online at https://www.mdpi.com/2072-6694/12/6/1469/s1, Figure S1. Spearman correlation analysis between relative telomere length and age. Relative lymphocyte telomere length (RLT) in peripheral blood and age of patients with gynecological cancer. Spearman correlation analysis in (A) patients age with ovarian cancer (N = 72) and absolute RLT value; (B) patients age with cervical cancer (N = 63) and absolute RLT value and (C) patients age with endometrial cancer (N = 87) and absolute RLT value. Figure S2. Hypothesis of Lymphocyte telomere length associated with host immunity. Hypothetical impact of telomere length on the host immune system. Long peripheral blood lymphocyte telomeres increase cytokines IL-2, IL-15, and IL-21, which are responsible for the proliferation of CD8+ T cells and good prognoses. Short telomeres cause an increase in IL-10, which suppresses the cellular immune response and is associated with poor prognosis. Table S1. Absolute value of RLT of OC, CC and EC.
