# Supplementary material for: Prognostic Value of Peripheral Blood Lymphocyte Telomere Length in Gynecologic Malignant Tumors
Source: Cancers (Basel). 2020 Jun 4;12(6):1469. doi: 10.3390/cancers12061469 (PMC7352644; doi:10.3390/cancers12061469)
Supplement: Supplementary file 1 [file cancers-12-01469-s001.pdf]

Supplemental Materials

Prognostic Value of Peripheral Blood Lymphocyte Telomere Length in Gynecologic Malignant Tumors

Kamrunnahar Shanta, Kentaro Nakayama, Masako Ishikawa, Tomoka Ishibashi Hitomi Yamashita, Seiya Sato, Hiroki Sasamori, Kiyoka Sawada, Sonomi Kurose, Hossain Mohammad Mahmud, Sultana Razia, Kouji Iida, Noriyoshi Ishikawa and Satoru Kyo

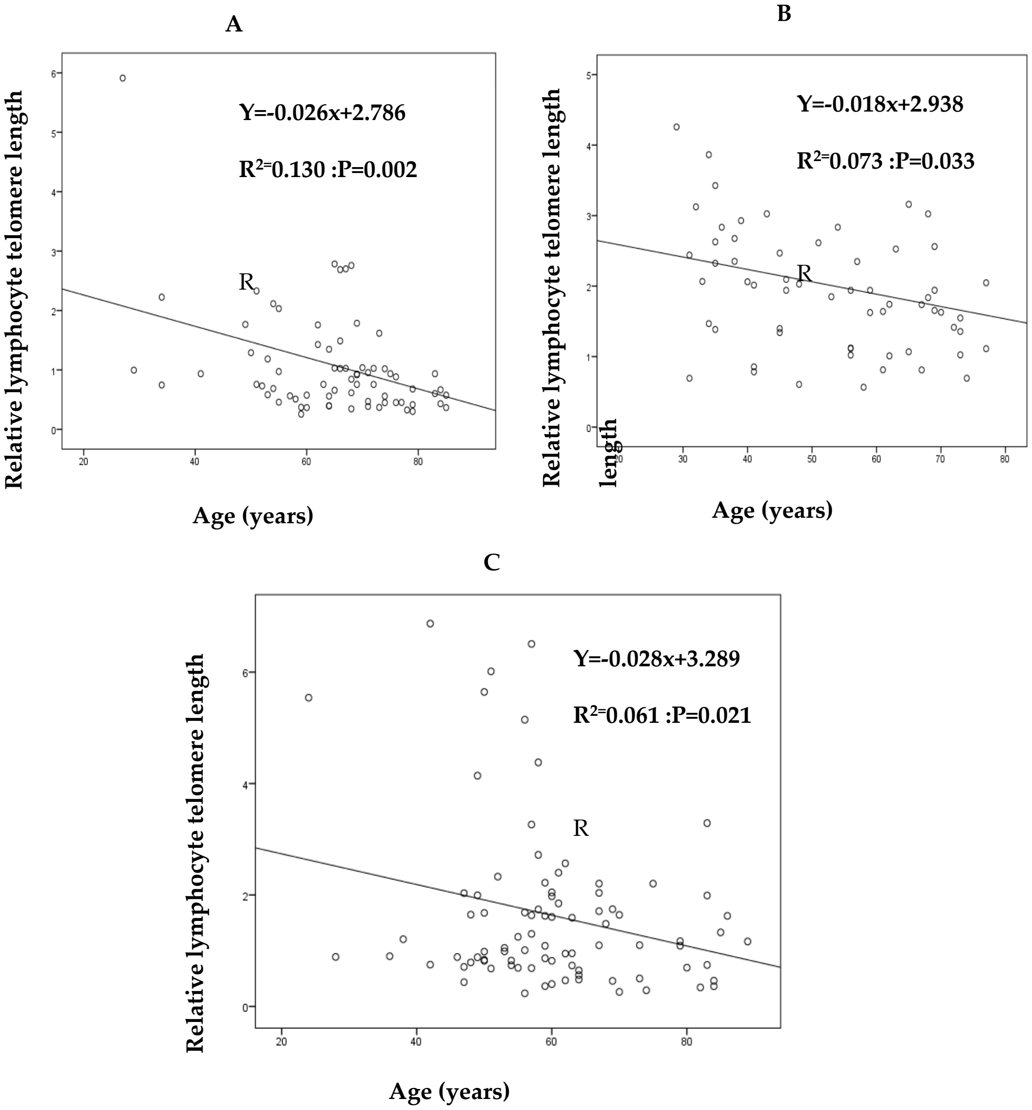

Figure S1. Spearman correlation analysis between relative telomere length and age. Relative lymphocyte telomere length (RLT) in peripheral blood and age of patients with gynecological cancer. Spearman correlation analysis in (**A**) patients age with ovarian cancer (N = 72) and absolute RLT value; (**B**) patients age with cervical cancer (N = 63) and absolute RLT value and (**C**) patients age with endometrial cancer (N = 87) and absolute RLT value.

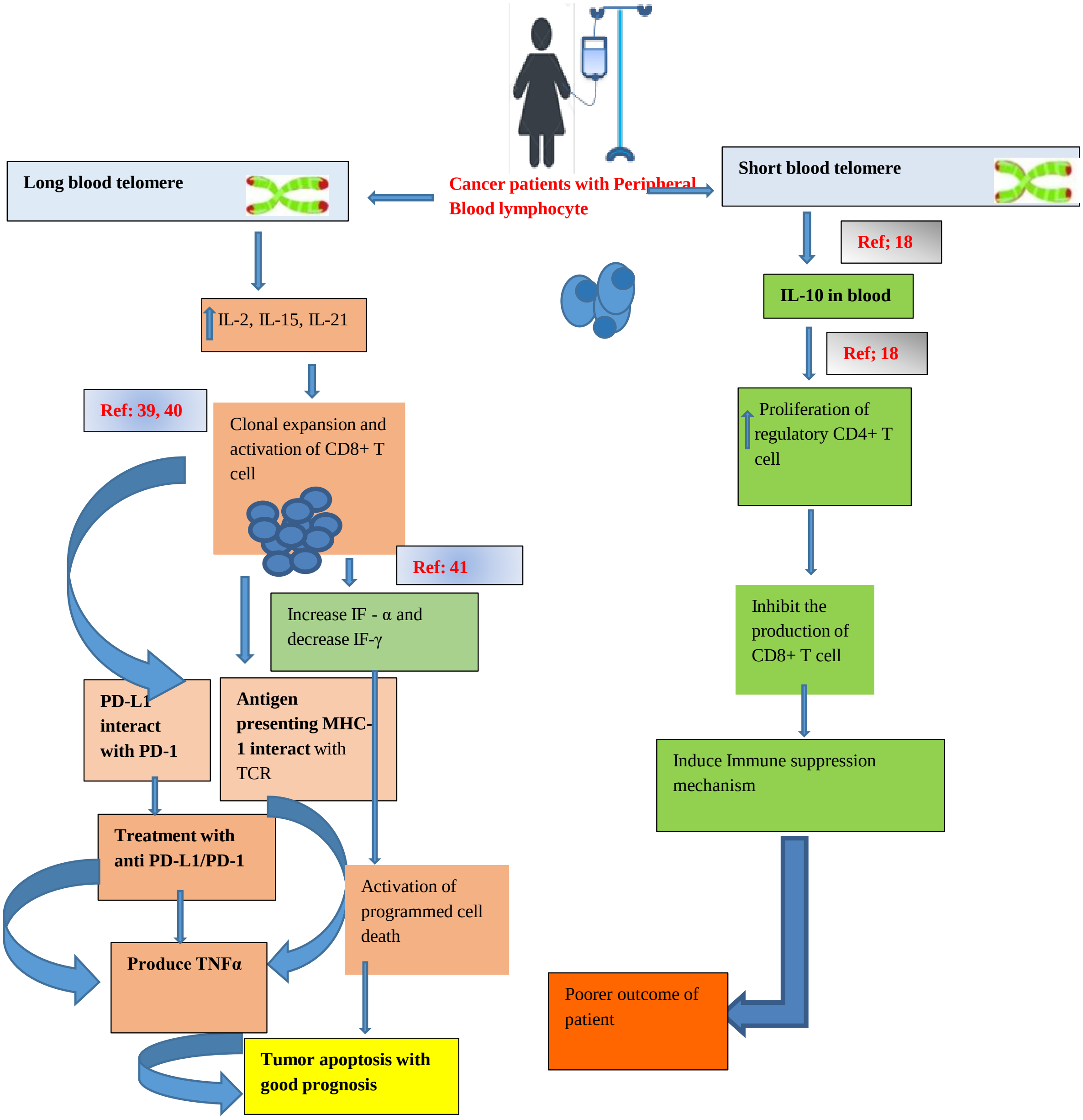

Figure S2. Hypothesis of Lymphocyte telomere length associated with host immunity. Hypothetical impact of telomere length on the host immune system. Long peripheral blood lymphocyte telomeres increase cytokines IL-2, IL-15, and IL-21, which are responsible for the proliferation of CD8+ T cells and good prognoses. Short telomeres cause an increase in IL-10, which suppresses the cellular immune response and is associated with poor prognosis.

Table S1. Absolute value of RLT of OC, CC and EC.

Case NO.	RLT of OC	Case NO.	RLT of CC	Case NO.	RLT of EC
1	2.329467	1	2.439637	1	0.361817
2	2.783050	2	2.324090	2	0.733736
3	2.688249	3	1.021012	3	0.457973
4	2.757447	4	1.024620	4	1.624505
5	3.944931	5	1.940683	5	0.988514
6	2.114036	6	1.068684	6	0.562529
7	0.414660	7	1.624505	7	0.743979
8	1.785919	8	1.628263	8	0.952638
9	0.301452	9	1.114709	9	1.647182
10	0.452712	10	1.940900	10	1.624505

11	0.448548	11	2.675855	11	5.540438
12	0.756109	12	2.060984	12	1.605846
13	0.368567	13	2.351096	13	1.301342
14	0.366021	14	1.848899	14	1.248331
15	0.365177	15	2.834970	15	1.741101
16	0.954842	16	1.639588	16	1.089249
17	0.395021	17	3.863745	17	1.685683
18	0.385108	18	2.348078	18	0.695762
19	0.395934	19	0.784580	19	1.086735
20	0.325335	20	1.356604	20	1.170128
21	0.255843	21	3.123870	21	0.363493
22	2.032610	22	0.605696	22	1.677912
23	1.037660	23	1.341022	23	1.205808
24	0.686184	24	0.565135	24	0.835088
25	1.289370	25	0.810378	25	1.709214
26	0.450625	26	2.046700	26	1.977028
27	0.678302	27	1.741101	27	1.635804
28	1.183724	28	1.940176	28	2.719485
29	0.972655	29	1.010493	29	2.037312
30	0.842842	30	2.065751	30	6.871653
31	0.562529	31	1.654811	31	2.329467
32	0.935191	32	3.758091	32	0.823591
33	1.025741	33	1.112136	33	0.983957
34	0.935191	34	2.013911	34	0.817902
35	0.430276	35	2.834970	35	2.400497
36	1.617015	36	1.385109	36	0.738840
37	0.665880	37	1.737083	37	0.260616
38	0.582367	38	1.547565	38	2.032610
39	0.882703	39	0.812252	39	1.990779
40	0.509328	40	1.414214	40	0.886791
41	0.935191	41	1.397970	41	2.046748
42	0.344681	42	2.614738	42	1.995384
43	0.756109	43	1.467472	43	1.745129
44	0.918064	44	0.692555	44	1.643381
45	1.427344	45	0.692555	45	1.164734
46	0.574349	46	2.928171	46	4.141060
47	0.601513	47	2.094588	47	0.749154
48	0.574349	48	3.160165	48	1.848899
49	1.347234	49	3.963202	49	0.289172
50	1.030492	50	3.024400	50	3.264058
51	0.469761	51	2.560900	51	3.289964
52	0.558644	52	3.024437	52	1.328686
53	0.754432	53	1.122462	53	1.482541
54	0.729863	54	2.525671	54	2.566853
55	1.765406	55	2.027919	55	5.145579
56	2.700700	56	4.247655	56	6.012846
57	0.370274	57	1.939810	57	2.203810
58	0.656712	58	2.626849	58	6.505990
59	5.912289	59	4.257481	59	2.219139
60	1.757267	60	1.836128	60	0.435275
61	2.224272	61	0.854607	61	0.483011
62	0.756109	62	2.467984	62	0.647671
63	0.995390	63	3.426336	63	0.948246
64	0.615572			64	0.888843
65	0.456916			65	0.234339
66	0.557354			66	0.790041
67	1.018656			67	0.679871
68	1.487958			68	0.340722

69	0.745700	69	1.591073
70	1.025741	70	0.467596
71	0.930880	71	0.501157
72	1.023374	72	2.203810
		73	0.400535
		74	0.708742
		75	0.687771
		76	1.096825
		77	1.098712
		78	1.009285
		79	0.461158
		80	0.692555
		81	4.379014
		82	0.901250
		83	0.864537
		84	1.049717
		85	5.643799
		86	0.817902
		87	0.882100
